# An *ANXA11* P93S variant dysregulates TDP-43 and causes corticobasal syndrome

**DOI:** 10.21203/rs.3.rs-3462973/v1

**Published:** 2023-10-19

**Authors:** Allison Snyder, Veronica H Ryan, James Hawrot, Sydney Lawton, Daniel M Ramos, Y Andy Qi, Kory Johnson, Xylena Reed, Nicholas L Johnson, Aaron W Kollasch, Megan Duffy, Lawren VandeVrede, J Nicholas Cochran, Bruce L Miller, Camilo Toro, Bibiana Bielekova, Jennifer S Yokoyama, Debora S Marks, Justin Y Kwan, Mark R Cookson, Michael E Ward

**Affiliations:** Neurogenetics Branch, National Institute of Neurological Disorders and Stroke; Center for Alzheimer’s and Related Dementias, National Institutes of Health; Neurogenetics Branch, National Institute of Neurological Disorders and Stroke; Neurogenetics Branch, National Institute of Neurological Disorders and Stroke; Center for Alzheimer’s and Related Dementias, National Institutes of Health; Center for Alzheimer’s and Related Dementias, National Institutes of Health; Intramural Bioinformatics, National Institute of Neurological Disorders and Stroke; Center for Alzheimer’s and Related Dementias, National Institutes of Health; Data Tecnica International LLC; Department of Systems Biology, Harvard Medical School; Cell Biology and Gene Expression Section, Laboratory of Neurogenetics, National Institute on Aging; Memory and Aging Center, Department of Neurology, Weill Institute for Neurosciences, University of California, San Francisco; HudsonAlpha Institute for Biotechnology; Memory and Aging Center, Department of Neurology, Weill Institute for Neurosciences, University of California, San Francisco; Undiagnosed Diseases Program, National Human Genome Research Institute; Neuroimmunological Diseases Section, National Institute of Allergy and Infectious Disease; Memory and Aging Center, Department of Neurology, Weill Institute for Neurosciences, University of California, San Francisco; Department of Systems Biology, Harvard Medical School, Boston; Office of the Clinical Director, National Institute of Neurological Disorders and Stroke; Cell Biology and Gene Expression Section, Laboratory of Neurogenetics, National Institute on Aging; Neurogenetics Branch, National Institute of Neurological Disorders and Stroke

**Keywords:** ANXA11, corticobasal syndrome, variant of uncertain significance, TDP-43

## Abstract

As genetic testing has become more accessible and affordable, variants of uncertain significance (VUS) are increasingly identified, and determining whether these variants play causal roles in disease is a major challenge. The known disease-associated Annexin A11 (ANXA11) mutations result in ANXA11 aggregation, alterations in lysosomal-RNA granule co-trafficking, and TDP-43 mis-localization and present as amyotrophic lateral sclerosis or frontotemporal dementia.

We identified a novel VUS in ANXA11 (P93S) in a kindred with corticobasal syndrome and unique radiographic features that segregated with disease. We then queried neurodegenerative disorder clinic databases to identify the phenotypic spread of ANXA11 mutations. Multi-modal computational analysis of this variant was performed and the effect of this VUS on ANXA11 function and TDP-43 biology was characterized in iPSC-derived neurons. Single-cell sequencing and proteomic analysis of iPSC-derived neurons and microglia were used to determine the multiomic signature of this VUS.

Mutations in ANXA11 were found in association with clinically diagnosed corticobasal syndrome, thereby establishing corticobasal syndrome as part of ANXA11 clinical spectrum. In iPSC-derived neurons expressing mutant ANXA11, we found decreased colocalization of lysosomes and decreased neuritic RNA as well as decreased nuclear TDP-43 and increased formation of cryptic exons compared to controls. Multiomic assessment of the P93S variant in iPSC-derived neurons and microglia indicates that the pathogenic omic signature in neurons is modest compared to microglia. Additionally, omic studies reveal that immune dysregulation and interferon signaling pathways in microglia are central to disease. Collectively, these findings identify a new pathogenic variant in ANXA11, expand the range of clinical syndromes caused by ANXA11 mutations, and implicate both neuronal and microglia dysfunction in ANXA11 pathophysiology. This work illustrates the potential for iPSC-derived cellular models to revolutionize the variant annotation process and provides a generalizable approach to determining causality of novel variants across genes.

## Introduction

With expanded access to genetic testing, the identification of novel genetic mutations and gene-disease associations has increased, but so too has the identification of variants of uncertain significance, which are the most commonly identified genetic change and increasing exponentially.^1^ At present, these represent a dead-end for both patients and researchers as current methods rely on a preponderance of clinical and experimental evidence as well as outdated *in silico* prediction models built from evolutionary conservation across species and magnitude of amino acid change to classify causality.^2^ Therefore, cellular and *in vivo* models are often employed to augment informatic predictions of pathogenicity, but applications of these models poorly generalize to other genetic variants within the same disease spectrum.

Annexin A11 (ANXA11) is a disease-associated protein that tethers RNA granules to lysosomes as they traffic in axons. The N-terminus of ANXA11 contains a low-complexity domain, which mediates phase transitions required for its condensation into membraneless RNA granules. Its C-terminus harbors four calcium-binding annexin domains, enabling regulated interactions of ANXA11 with negatively-charged lysosomal membranes. Disease-associated mutations in both N-terminal and C-terminal domains of ANXA11 have been described, which alter its phase separation properties and disrupt its RNA-lysosome tethering function.^3–6^

The number of diseases associated with variants in ANXA11 has broadened substantially since 2006, when a genome wide association study first implicated it as a risk gene for autoimmune disease and sarcoid.^7^ In 2017, multiple ANXA11 mutations were found to cause genetic forms of amyotrophic lateral sclerosis.^8^ These cases exhibited both TDP-43 and ANXA11 aggregates in the CNS.^8^ Subsequent independent studies identified these and other ANXA11 variants in association with familial forms of frontotemporal dementia, fitting the rubric of genes causing a spectrum of disease from amyotrophic lateral sclerosis to frontotemporal dementia.^9^ In 2021, ANXA11 variants were found to cause an inclusion-body myopathy-like syndrome affecting limb-girdle, axial, and distal leg musculature.^10^ Clinical phenotypes within the described inclusion-body myopathy kindreds included both amyotrophic lateral sclerosis/frontotemporal dementia features as well as two cases of frontotemporal dementia, indicating that ANXA11 mutations can cause a variety of distinct phenotypes within the frontotemporal dementia/amyotrophic lateral sclerosis/inclusion body myopathy disease spectrum.

Here we describe a family with a P93S variant of uncertain significance (VUS) in ANXA11 who present clinically with corticobasal syndrome, a novel clinical phenotype not previously associated with this gene. We further identified additional patients with mutations in ANXA11 and similar clinical presentations from a large neurodegenerative disease cohort, thereby establishing corticobasal syndrome as part of the phenotypic spectrum of ANXA11 mutations. Using iPSC-based models, coupled with proteomic, transcriptomic, and microscopy readouts, we show that the P93S ANXA11 variant alters key functional properties of ANXA11. Expression of the P93S ANXA11 variant causes abnormalities in TDP-43 expression and function, which are hallmarks of amyotrophic lateral sclerosis/frontotemporal dementia pathophysiology. Finally, the P93S ANXA11 variant substantially alters the transcriptome and proteome of microglia, suggesting that ANXA11 may play crucial roles in glia in addition to its known importance in neuronal biology.

## Materials and methods

### Clinical evaluation of the P93S kindred

Members of the P93S kindred were seen under the NIH IRB approved protocol entitled “Investigating Complex Neurodegenerative Disorders related to Amyotrophic Lateral Sclerosis and Frontotemporal Dementia” and consent was obtained in accordance with the Declaration of Helsinki. Enrolled participants underwent comprehensive assessments including history and neurological examination, neuropsychological testing, and caregiver questionnaires. Motor rating was captured using ADPM wearable technology. Electromyography and nerve conduction study were performed. MR imaging was obtained on a 3T scanner including sagittal 3D T1-weighted, fat suppressed axial T2-weighted, fat suppressed coronal T2-weighted, axial FLAIR, and axial DTI images of the brain and sagittal T1-weighted, sagittal STIR, sagittal T2-weighted, axial T2-weighted, and axial s3D MEDIC-MT, postcontrast sagittal T1-weighted, and postcontrast axial 3D T1-weighted images of the spine. Lumbar puncture was performed in the upright, seated position with insertion of an atraumatic Sprotte needle in the L4/5 space and analyzed for cell counts and differential, protein, glucose, oligoclonal bands and IgG index. Additional exploratory CSF analysis included absolute and relative quantification of CSF immune cell subsets using multicolored flow cytometry and proteomic inflammatory biomarkers were performed according to previously published protocols.^11^

### Clinical spectrum of ANXA11 mutations

The NIH Undiagnosed Diseases Program as well as the University of California San Francisco Memory and Aging Center cohorts were queried for cases harboring ANXA11 mutations using a minor allele frequency filter of 1 in 10,000 in gnomAD. Identified cases were then reviewed for diagnosis and clinical features by board-certified neurologists (AS, JK, LV).

### Cell culture and differentiation

The NIH Intramural research program policies were followed for the procurement and use of WTC11 line iPS cells from the Coriell cell repository, which were derived from a 30-year-old male without known neurological disease. Culture procedures have been described previously.^12^ Briefly, iPS cells were grown on tissue culture dishes precoated with Matrigel (Corning #354277) in Essential 8 medium (Thermo #A1517001), replaced every one to two days as needed. Accutase (Thermo #A1110501) was used for cell dissociation and passaging. Chroman-1 (MedChemExpress Catalog #HY-15392) supplementation was used to promote survival upon thawing, passaging, and other cell line modifications until cell colonies contained a minimum of five cells. The human iPS cells used in this study were previously engineered to express mouse neurogenin-2 (mNGN2) with a doxycycline-inducible promoter integrated in the AAVS1 and CLYBL promoter safe harbor sites for differentiation into cortical-like neurons or transcription factors MAFB, IRF8 and CEBPA in the AAVS1 and SPI1, CEBPB, and IRF5 in the CLYBL safe harbor sites for differentiation into microglia-like cells.

Plasmids for viral transduction into iPS cells were generated using the pLEX lentiviral vector and ligation cloning to generate fluorescently labeled vectors for the overexpression of ANXA11 based off previously published constructs.^3^ Following sequence confirmation, the plasmids were transfected into Lenti-X HEK cells using Lipofectamine^™^ 3000 (Invitrogen). Twenty-four hours after transfection, viral boost reagent was added. Medium was collected after 72 hours and concentrated using Lenti-X concentrator (Takara Bio) overnight before being aliquoted and stored at −80°C. After dissociation and singularization using StemPro Accutase^™^ (Gibco), viral aliquots were delivered to iPS cells in E8 medium with Chroman-1. Cells were monitored for transduction efficiencies with a goal of at least 80% of cells expressing the fluorescent green construct, equal across lines. Media was changed to E8 medium and iPS cells were expanded and frozen for use in differentiations and downstream experiments.

Neuronal differentiation followed previously published protocols.^12^ Briefly, cells expressing doxycycline-inducible mNGN2 were plated on Matrigel-coated plate in neuronal induction medium: DMEM/F12 (Thermo #11330032) supplemented with N2 supplement (Thermo #17502048), Non-essential amino acids (Thermo #11140050), Glutamax (Thermo #35050061), 2μg/mL doxycycline (Sigma #D9891) and 50nM Chroman-1 on day zero. Fresh induction medium without Chroman-1 was added after PBS washes on days one and two. On day three, cells were dissociated using Accutase, counted, and replated onto poly-L-ornithine (PLO)-coated plates in neuronal maturation medium containing 2μg/mL doxycycline. On day four, the medium was changed after a PBS wash. Thereafter, a half medium change was conducted twice weekly. All neuron experiments were conducted using day 15 neurons unless otherwise specified.

Microglial differentiation followed previously published protocols.^13^ Briefly, cells expressing the six transcription factors were plated on a dual Matrigel- and PLO-coated tissue culture dish in Essential 8 medium with doxycycline and Chroman-1 on day zero. On day two, medium was changed to Advanced DMEM/F12 (Thermo #12634010) with 2μg/mL doxycycline, GlutaMAX (Thermo #35050061), 100 ng/mL Human IL-34 (Peprotech #200 – 34) and 10 ng/mL Human GM-CSF (Peprotech #300–03) after PBS washes. On day four, medium was changed with the addition of 50 ng/mL Human M-CSF (Peprotech #300 – 25), 50 ng/mL Human TGF-β1 (Peprotech #100–21C), and 50 ng/mL Mevalonolacton (Sigma-Aldrich #M4667–1G), and antibiotic/antimycotic (Gibco #15240–062). Thereafter, half medium changes were conducted twice weekly. All microglial experiments were performed on day 15 unless otherwise specified.

### Lysosome colocalization

WTC11 iPS cells with the mNGN2 construct overexpressing wildtype and P93S mutant ANXA11 were transduced with a lentiviral vector containing LAMP1 fluorescently labeled with mApple as described in cell culture methods above. Following transduction, iPS cells were differentiated into iPSC-derived neurons as spheroids. For spheroid differentiation, 10,000 iPS cells were resuspended in 20 μL neuronal induction medium per sphere after splitting iPS cells with Accutase. 20 μL of the iPS cell suspension was added to a well of an ultra-low attachment round bottom 384-well plate (Corning) coated with anti-adherence solution (Life Technologies) for 1 hour and washed with PBS 3 times. Cells were left to sit for 5 min before centrifuging at 150 RCF for 2 min to promote cells aggregate. The next day, 60 μL neuronal induction medium was added. The third day after addition of doxycycline, spheres were pipetted up using a wide-bore, low-attachment pipette tip and plated on an 8-chamber glass bottom slide (Ibidi) coated with poly-L-ornithine followed by a 2-h coating with 15 μg/mL laminin and prepped with 250uL neuronal maturation medium. Spheroids were allowed to grow for four more days with a full medium change the day after replating and a half medium change every three days thereafter. On day seven after doxycycline, iPSC-derived neurons were imaged using Nikon spinning disc equipped with a 60x water immersion objective. Live cell imaging was focused on regions of the well with clear growth cones and relatively sparse axons to reduce the number of intersecting axons in the captured image area. Eight images were captured per well with four replicates per condition. NIS Elements general analysis 3 was used to quantify red and green puncta corresponding to lysosomes and ANXA11 granules respectively. Total counts of lysosomes colocalized with ANXA11 were calculated as well as mean counts per well. Data was assessed for normality using Shapiro-Wilk test and unpaired t-test was applied to well means to determine significance. Data was visualized in GraphPad Prism 9.5.1.

### Quantification of neuritic RNA

RNAscope Assay (Advanced Cell Diagnostics) was used to assess delivery and distribution of RNA in iPSC-derived neurons according to the manufacturer’s protocol.^14^ Briefly, iPSC-derived neurons overexpressing wildtype and P93S mutant ANXA11 were plated on an 8-chamber glass bottom slide (Ibidi) at a density of 12,000 cells per well in 250 μL medium. On day 15 after doxycycline, cells were fixed with 4% paraformaldehyde for 10 minutes at room temperature, then washed three times with PBS. Cells were then sequentially dehydrated in increasing concentrations of ethanol and frozen at −20°C overnight. The following day cells were rehydrated before treatment with protease. The cells were probed for beta-actin in the HybEZ oven at 40°C for two hours. Cells were incubated with Amp1-FL, Amp2-FL, Amp3-FL and Amp4-FL solutions sequentially prior to Hoechst nuclear counterstaining. Cells were imaged immediately for identification of beta-actin RNA puncta. The following day, cells were incubated with anti-tau (R&D AF3494) and anti-H4A3 (DSHB AB 2296838) antibodies in PBS at a 1:1,000 dilution overnight at 4°C. The next day, cells were washed three times with PBS before being incubated with anti-goat and anti-rabbit secondary antibodies (Biotium #20016 and #20098) for thirty minutes at room temperature and reimaged. Imaging was performed on the Nikon spinning-disk confocal microscope (Nikon Eclipse T1) using a 60× water immersion objective lens. Eight images were captured per well with eight replicates per condition. NIS Elements general analysis 3 was used to quantify RNA puncta. Total neuritic RNA puncta per total RNA puncta were calculated as well as means per well. Data was assessed for normality using Shapiro-Wilk test and unpaired t-test was applied to well means to determine significance. Data was visualized in GraphPad Prism 9.5.1.

### TDP-43 immunocytochemistry

Immunocytochemistry was performed on 96-well plates (Perkin Elmer) or μ-Slide glass bottom slides (Ibidi). On day 15 post differentiation, cells were fixed using 4% paraformaldehyde for 15 minutes at room temperature. Following three PBS washes, cells were permeabilized using 0.1% Triton-X100 for 10 minutes at room temperature. Cells were then blocked in 2% bovine serum albumin for 60 minutes. Primary antibody incubation was performed overnight at 4°C at 1:500 concentration of anti-TDP-43 antibody (Abcam ab254166). Immunofluorescence was detected with fluorochrome-conjugated secondary antibodies CF640 donkey anti-mouse (1:1,000) for detection of TDP-43. Finally, nuclei were counterstained with Hoechst (2 μg/mL, Thermo Fisher Scientific no. 62248). Images were acquired on an inverted Nikon spinning-disk confocal microscope (Nikon Eclipse T1), using a 60× 1.40 NA oil-immersion objective. Twelve images were captured per well with four replicates per condition. Quantification of nuclear TDP signal was performed using CellProfiler. Total mean intensities of nuclear TDP-43 signal were calculated as well as means per well. Data was tested for normality using Shapiro-Wilk test and unpaired t-test was applied to well means to determine significance. Data was visualized in GraphPad Prism 9.5.1.

### Detection of cryptic exons

HCR FISH custom probes were designed using Molecular Instruments Custom Probe Design Tool to target native and cryptic exons in two genes known to be associated with TDP-43 loss of function: UNC13A and STMN2 (Supplementary material). Ability to detect cryptic RNA was first tested in iPSC-derived neurons expressing a catalytically dead Cas9 fused to a KRAB transcriptional repression domain to allow inhibition of gene transcription with control non-targeting guide and a guide to knockdown TDP-43 expression on day seven after doxycycline. HCR FISH was performed according to the manufacturer’s protocol.^15^ Briefly, iPSC-derived neurons overexpressing wildtype and P93S mutant ANXA11 were plated on a 384-well plate (Corning) at a density of 3,000 cells per well in 100 μL medium. On day 15 after doxycycline, cells were fixed with 4% paraformaldehyde for 10 minutes at room temperature, then washed three times with PBS. Fixed cells were permeabilized with 70% ethanol overnight at −20°C. Ethanol was removed the following day and cells were washed twice with 2x SSC buffer (Molecular Instruments). Before adding the probe solutions, cells were incubated in warm probe hybridization buffer for 30 minutes at 37°C. Probe solutions were prepared with 0.8 pmol of each probe set in probe hybridization buffer. Cells in probe solutions were hybridized overnight at 37°C. The following day, cells were washed four times with warm probe wash buffer. Cells were then washed twice with 5x SSCT at room temperature. Samples were amplified for 30 minutes at room temperature in amplification buffer while hairpin solutions were prepared. 12 pmol of each hairpin one and two were heated to 95°C for 90 seconds then cooled to room temperature without exposure to light for 30 minutes. Cooled hairpins were added together in amplification buffer at room temperature before being added to the cells. Samples were incubated at room temperature overnight without exposure to light. The next day, hairpin solutions were removed, and cells were washed five times with 5x SSCT. Cells were incubated with Hoechst in PBS for nuclear counterstaining at a 1:10,000 dilution for five minutes at room temperature. Cells were washed three times with PBS and stored at 4°C until being imaged. Cells were imaged with a Nikon spinning disk confocal on a 60x water immersion objective lens using a random imaging job with the nucleus as the plane of focus. NIS Elements general analysis 3 was used to quantify cryptic exons. Total cryptic exons per cell were calculated as well as means per well. Data was assessed for normality using Shapiro-Wilk test. To determine significance, unpaired t-test was applied to data passing tests of normality and Mann-Whitney test was applied to data not passing tests of normality. Data was visualized in GraphPad Prism 9.5.1.

### Single cell RNA sequencing

iPSC-derived neurons and microglia on day 15 post differentiation were washed with PBS once and then washed twice with PBS + 0.5 mM EDTA before adding 1 mL of Papain at 10 units/mL (Worthington) in TrypLE. Cells were incubated for five minutes at 37°C until cells were starting to physically detach. Enzyme mixture was aspirated, and cells were resuspended in trituration medium (BrainPhys medium supplemented with Chroman-1 and 33ug/mL DNase I (Worthington)) using a p1000 pipette tip until single cells are visible under light microscopy. Cells were collected and centrifuged at 200 ×g for 5 minutes at room temperature. Supernatant was aspirated and cells were resuspended in trituration medium with ovomucoid papain inhibitor at 10 mg/mL (Worthington). Cells were centrifuged at 200 ×g for 5 minutes at room temperature. Cells were then resuspended in BrainPhys medium and counted. Cells were then washed three times with PBS + 0.04% Bovine Serum Albumin using a wide-bore tip p1000 pipette. Cell solutions were counted using an automated cell counter (Countess II). Final cell solution was recounted and diluted to a target concentration of 1000 cells/μL and placed on ice.

Single cells were isolated using the Chromium Connect platform where 5000 single cells were targeted for capture from each sample. Single cell expression libraries were constructed using the Chromium Next GEM Automated Single Cell 3’ Library and Gel Bead Kit v3.1 and the Chip G Automated single cell kit (10x Genomics). Libraries were pooled and sequencing was completed on an Illumina NextSeq 550 system using a NextSeq 150 Cycle Hi-Output v2.5 kit (Illumina #20024907), generating a total of 400 million reads for an estimated sequencing depth of 40,000 reads per cell.

Raw sequencing data were aligned using bcl2fastq v2.20.0 and counts tables were generated using Cell Ranger software v7.0.0 (10x Genomics). Normalization, integration, and clustering analysis of these two scRNAseq datasets was completed using the Seurat package (version 4.3.0) in R as previously described.^16,17^ Data were filtered to a minimum of 800 UMIs per cell and 200 genes per cell. Cells with more than 6000 genes or greater than 10% mitochondrial reads were excluded, and a minimum 0.8 ratio of the base 10 logs of genes per cell and UMIs was required per cell. UMAP clustering was performed using the Louvain algorithm at a resolution of 0.2 and included 32 principal components. The neuronal cluster was identified by expression of TUBB3 and DCX, however PHOX2B positive neurons were not included in pseudobulk differential expression analysis. The microglial cluster was characterized by expression of APOE and AIF1. Pseudobulk analysis was completed using DESeq2 v1.38.0.^18^ The linear model was used to account for batch effects in the data. The ashr shrinkage algorithm was applied to penalize high log fold changes of minimally expressed transcripts.

### Mass spectrometry-based proteomics

Sample preparation for proteomics was performed according to previously published protocols and included an automated pipeline for protein assay, capture and digestion.^19,20^ Briefly, iPSC-derived neurons and microglia were harvested on day 15 from 6-well plates with six replicates per condition. Cells were washed with ice-cold PBS prior to collection using a high-percentage detergent lysis buffer (50 mM HEPES, 50 mM NaCl, 5 mM EDTA 1% SDS, 1% Triton X-100, 1% NP-40, 1% Tween 20, 1% deoxycholate and 1% glycerol) supplemented with complete protease inhibitor cocktail at 1 tablet/10 mL ratio. The cell lysate was reduced by 10 mM dithiothreitol for 30 min at 60°C and alkylated using 20 mM iodoacetamide for 30 min in the dark at room temperature. The denatured proteins were captured by hydrophilic magnetic beads. Tryptic on-beads digestion was conducted for 16 h at 37°C. Tryptic peptides were dehydrated and resuspended in a 2% acetonitrile / 0.4% trifluoroacetic acid solution and normalized to a concentration of 0.2 mg/mL for liquid chromatography-mass spectrometry (LC-MS) analysis.

For the LC-MS analysis, we employed a direct-data-independent acquisition (dDIA) single-shot approach. Briefly, sample peptides were separated on a nano LC and subsequently analyzed on an Orbitrap Eclipse MS. A linear 120 min LC gradient with 2–35% solvent B (0.1% formic acid, 5% dimethyl sulfoxide in acetonitrile) were used on 75 μm by 500 mm LC column with 2 μm C_18_ particle (Thermo Scientific, Cat # ES903). The MS1 scan was set at 12,000 resolutions with an auto injection time; the MS2 scan isolation windows were set to 8 m/z (400–1,000 m/z range), 3 s cycle time, and 30,000 resolution. For protein annotation, MS RAW files were database searched using dDIA approach in Spectronaut (v14.1, Biognosys, Inc) against a human proteome reference containing 20,586 reviewed protein entries (Uniprot-Human-Proteome_UP000005640). The raw intensity of quantified proteins was medium normalized across all samples from the same condition.

Normalized peptide abundances were pedestalled by two then base two log transformed. Cyclic loess normalization was applied to correct for differences in distribution. Mean abundance per protein was modeled by the coefficient of variation observed per protein per condition. Modeling non-linear fits identified 9.5 as the value at which linearity is lost and thus applied as the filter. ANOVA testing followed by post-hoc testing was used to identify differentially expressed proteins.

### Data availability

The data that support the findings of the clinical spectrum of ANXA11 mutations can be found at https://adknowledgeportal.synapse.org/Explore/Studies/DetailsPage/StudyDetails?Study=syn25686496 but further clinical information will not be made publicly available to protect the privacy of research participants. The code for the analysis of single cell sequencing can be found at: https://github.com/NIH-CARD/ANXA11_novel_variant.git and mass-spectrometry based proteomic findings are openly available at PRIDE. The data that support the remaining findings in this study are available from the corresponding author, upon request.

## Results

### Clinical evaluation of the P93S kindred

**Figure 1:**
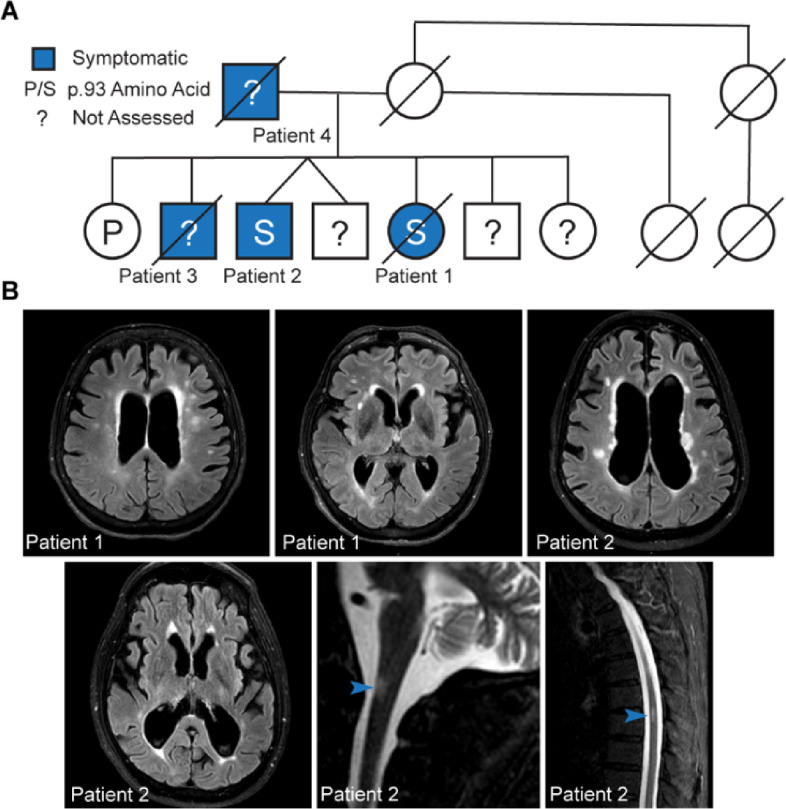
P93S kindred. **(A) Pedigree of P93S family.** Genetic testing is available from three individuals; the position 93 amino acid is indicated by letter and unavailable testing is indicated by a question mark. Symptomatic individuals, indicated in blue include patients one, two, three, and four. (**B) Representative MRI findings.** T2-weighted FLAIR MRI images from patients one and two demonstrating central-predominant atrophy in a frontoparietal distribution and prominent white matter hyperintensities in the cerebral cortex and white matter hyperintensities on spinal cord images from patient two, indicated by arrowheads.

We identified four individuals across two generations with a corticobasal-like syndrome harboring a novel variant in the *ANXA11* gene. Whole exome sequencing of the proband, one affected sibling, and one unaffected sibling revealed a variant of uncertain significance in ANXA11 c.277C > T p.P93S (rs753295755) in exon 4 that segregated with disease (Fig. 1A). This missense variant occurs in the intrinsically disordered low complexity domain at the N-terminus of the protein. All affected family members present with a slowly progressive asymmetric corticobasal-like syndrome and white matter abnormalities on MRI (Fig. 1B). Both the proband and her brother suffered from chronic pain as well. Cerebrospinal fluid analysis of the proband and her brother reveal no significant abnormalities in the absolute and relative numbers of CSF cells of adaptive immunity. However, both patients had elevated proportions of innate lymphoid cells, either in CSF (proband) or blood (brother) and the proband had also higher proportion of CSF granulocytes. Both patients had highly elevated CSF NFL, a marker of neuro-axonal injury, and CHIT3L1 a marker associated with intrathecal activation of innate immunity, expressed mainly in astrocytes and macrophages/microglia. Both subjects had also slightly elevated CSF IgG levels but only the proband had increased IgG index. Consistent with normal levels of CSF T cells, CSF levels of T cell activation marker sCD27 were also normal (Table 1; Supplementary Material).

### Clinical spectrum of ANXA11 mutations

To further characterize the spectrum of clinical phenotypes and ANXA11 mutations, we queried neurodegenerative disorders clinics for additional cases. We identified three additional cases of corticobasal syndrome in carriers of previously identified ANXA11 mutations or variants of uncertain significance (Table 1), along with the previously described clinical phenotypes such as behavioral variant frontotemporal dementia, amyotrophic lateral sclerosis/frontotemporal dementia, and amyotrophic lateral sclerosis cases (Table 1; Supplementary Material).

In general, this clinical presentation was typified by slowly progressive disease, in contrast with ANXA11-associated amyotrophic lateral sclerosis cases. CNS imaging studies revealed white matter atrophy in a posterior frontoparietal distribution with or without T2 FLAIR hyperintensities. Taken together, this kindred and other ANXA11 mutation carrier cases expand the known clinical spectrum of mutations in ANXA11 to include a corticobasal syndrome presentation.

### Computational assessment of ANXA11 P93S variant

**Figure 2:**
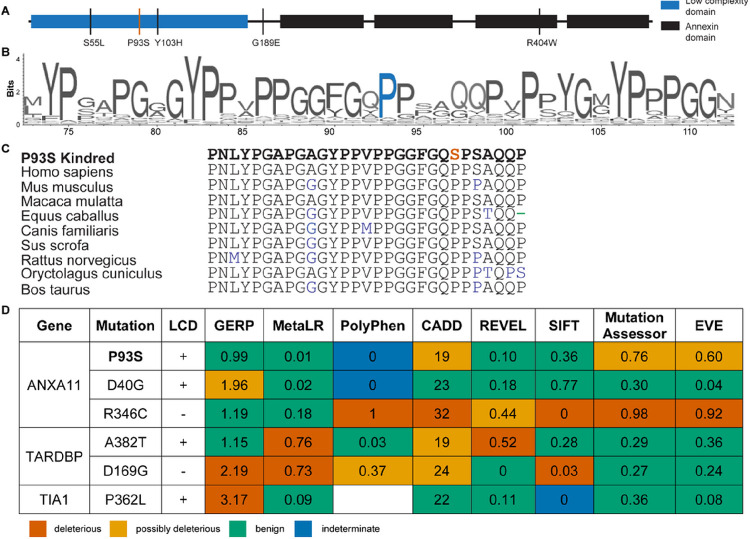
ANXA11 Structure. **(A) Schematic of ANXA11.** Low complexity domain is shown in blue and annexin domains are shown in black. The P93S variant is identified by a red line; remaining variants/mutations are indicated by black lines. (**B) and (C) ANXA11 sequence conservation.** VarSite illustration of sequence conservation with the proline in position 93 indicated in blue **(B)** and sequence conservation of annexin A11 across vertebrate species **(C)** with the P93S variant indicated in red. (**D) Poor performance of *in silico* variant prediction models for mutations in LCDs.** P93S VUS with other known pathogenic mutations in common amyotrophic lateral sclerosis/frontotemporal dementia associated genes with LCDs are shown. The predictions of pathogenicity by color: predicted benign in green, moderate in orange, pathogenic in red, and indeterminate in blue. GERP score considered deleterious >2. MetaLR score ranges from 0 benign to 1 deleterious. PolyPhen score considered deleterious >0.446. CADD score considered deleterious >30. REVEL score ranges from 0 benign to 1 deleterious. SIFT score considered deleterious <0.05. Mutation Assessor score ranges from 0 benign to 1 deleterious. EVE score considered potentially deleterious >0.5 and deleterious >0.7.

The P93S VUS is located within the low complexity domain (LCD), a region of ANXA11 in which previously identified amyotrophic lateral sclerosis/frontotemporal dementia mutations reside (Fig. 2A). The proline at position 93 of ANXA11 is highly conserved in land mammals, suggesting a potential physiological importance to protein function (Fig. 2B–C). However, further characterization of potential pathogenicity of the P93S variant using a set of *in silico* models were inconclusive (Fig. 2D), with only some models suggesting even moderate pathogenicity (e.g. MutationAssesor, EVE, CADD), while most others were suggestive of a benign change.^21–29^ The variant is extremely rare in population databases, with identification of only one carrier of unknown age that overlaps between gnomAD v3.1.2 (76,156 genomes) and TOPMed Bravo freeze 10 (150,899 genomes) and one carrier, which may be the same individual, of age 35–40 in exomes (125,748 total) from gnomAD v2.1.1. This rarity is consistent with pathogenicity as many dominant and established pathogenic neurodegeneration-associated variants are present in population databases with even higher counts, e.g. PSEN1 A79V (5 counts in gnomAD v3.1.2) or MAPT R406W (4 counts in gnomAD v2.1.1). Collectively, these findings hint that P93S may be pathogenic, but in isolation are insufficient to assign definitive causality to this novel variant.

### The P93S variant alters core functions of ANXA11

**Figure 3:**
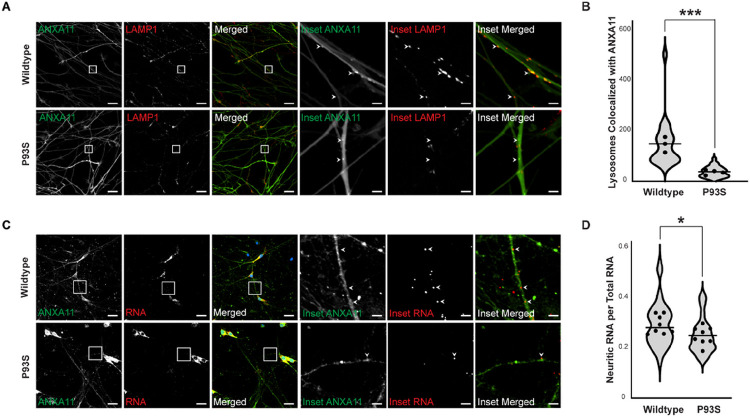
Functional impact of P93S variant. **(A) and (B) Decreased colocalization of lysosomes in mutant ANXA11 neurons. (A)** Representative images of iPSC-derived neurons expressing wildtype and mutant ANXA11 (green) and LAMP1 lysosomal marker (red) showing colocalization of lysosomes with ANXA11 puncta indicated by arrowheads, scale bar = 25 μm, inset scale bar = 4 μm. **(B)** Quantification of number lysosomes with ANXA11, well mean indicated by dot, horizontal line indicates median, p = 0.0009. (**C) and (D) Decreased neuritic RNA in mutant ANXA11 neurons. (C)** Representative images of fixed iPSC-derived neurons expressing wildtype and mutant ANXA11 with in situ hybridization probes for β-actin RNA using RNAscope to identify neuritic RNA indicated by arrowheads, scale bar = 25 μm, inset scale bar = 4 μm. **(D)** Quantification of proportion of neuritic RNA over total RNA, well mean indicated by dot, horizontal line indicates median, p = 0.03.

Given the contradictory above *in silico* predictions of the P93S variant, we turned to cellular models to characterize its effects on protein function. Prior studies show that ANXA11 disease-associated mutations can disrupt the interaction of ANXA11 with lysosomes.^3^ Therefore, we tested whether the P93S variant altered ANXA11 co-localization with lysosomes by imaging iPSC-derived human neurons infected by lentiviruses expressing either wildtype or P93S ANXA11 and the LAMP1 lysosome marker (Fig. 3A). We calculated the number of lysosomes colocalizing with ANXA11 granules and see that the P93S mutation results in a 75% decrease in the association of the ANXA11 granules with lysosomes (*mean* = 143.8 vs 35.34, *P* = 0.0009; Fig. 3B; Supplementary Fig. 1A-B). ANXA11 tethers RNA granules to lysosomes during axonal transport. We examined whether the P93S variant altered axonal RNA trafficking in iPSC-derived neurons using RNAscope, an RNA in situ hybridization technique (Fig. 3C). Expression of the P93S variant reduced axonal beta-actin RNA abundance compared to wild-type ANXA11 by 15% (*mean* = 0.2816 vs 0.2398, *P* = 0.03; Fig. 3D). These findings indicate that P93S alters the ability of ANXA11 to interact with lysosomes, consequently impeding delivery of RNA to distal neuritic compartments.

### The P93S variant causes nuclear clearance and TDP-43 dysfunction

**Figure 4:**
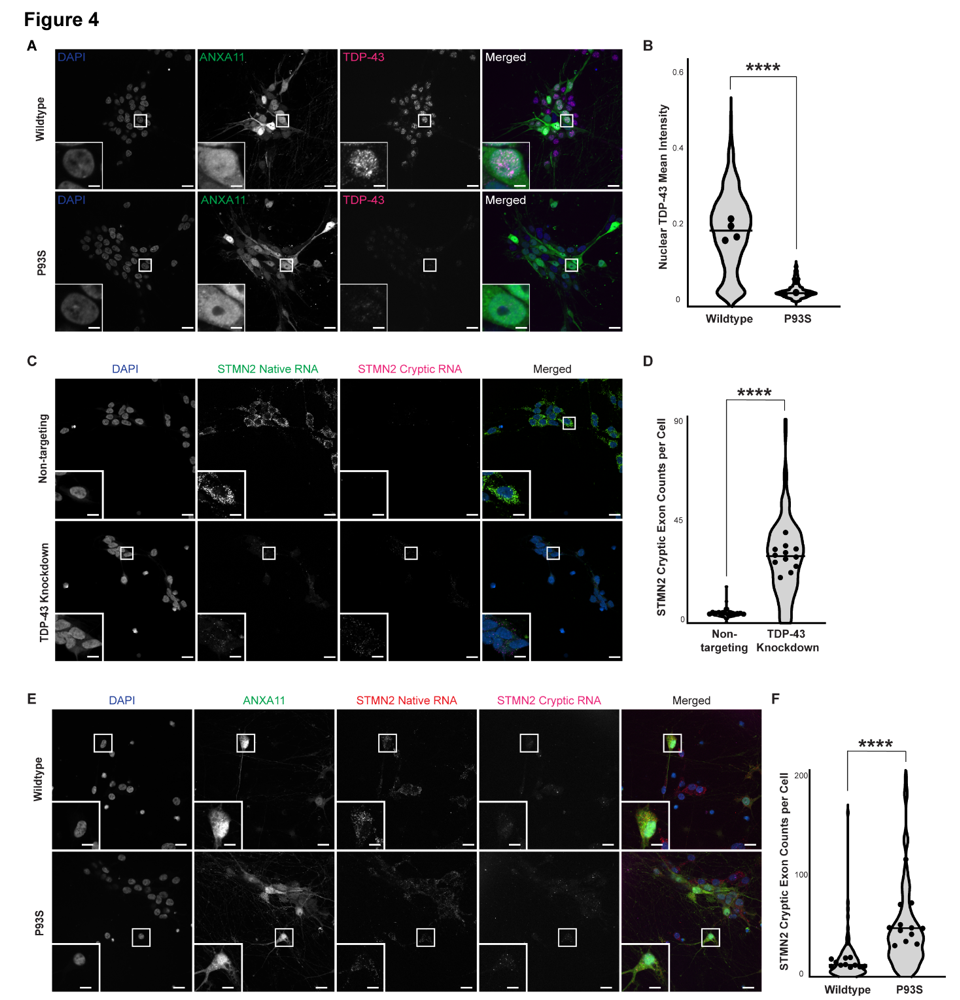
Decreased nuclear TDP-43 and formation of cryptic exons. (A) and (B) Decreased nuclear TDP-43 in mutant ANXA11 neurons. **(A)** Representative images of fixed iPSC-derived neurons expressing wildtype and mutant ANXA11 (green) stained with TDP-43 (magenta) and Hoechst nuclear counterstaining (blue) demonstrating nuclear clearing of TDP-43 in mutant ANXA11 cells, scale bar = 25 μm, inset scale bar = 1.75 μm. **(B)** Quantification of mean TDP-43 intensity in wildtype compared to mutant ANXA11, well mean indicated by dot, horizontal line indicates median, p <0.0001. (**C) and (D) Detection of STMN2 cryptic exon formation in TDP-43 KD neurons. (C)** Representative images of fixed iPSC-derived CRISPRi neurons with control non-targeting and TDP-43 knockdown guides demonstrating detection of native STMN2 RNA (green) and cryptic RNA (magenta) using HCR FISH probes with Hoechst nuclear counterstaining (blue), scale bar = 25μm, inset scale bar = 4 μm. **(D)** Quantification of cryptic exon counts per cell in non-targeting and TDP-43 knockdown cells for STMN2, well mean indicated by dot, horizontal line indicates median, Mann Whitney p<0.0001. **(E) and (F) Increased STMN2 cryptic exon formation in mutant ANXA11 neurons. (E)** Representative images of fixed iPSC-derived neurons expressing wildtype and mutant ANXA11 (green) with HCR FISH probes for native STMN2 RNA (red) and cryptic RNA (magenta) with Hoechst nuclear counterstaining (blue), scale bar = 25 μm, inset scale bar = 4 μm. **(F)**Quantification of cryptic exon counts per cell in wildtype and mutant ANXA11 cells for STMN2, well mean indicated by dot, horizontal line indicates median, p<0.0001.

Patients with amyotrophic lateral sclerosis/frontotemporal dementia due to ANXA11 mutations develop hallmark pathological features of TDP-43 mislocalization, including its loss in the nucleus and aggregation in the cytoplasm.^8,30^ We evaluated our P93S and wildtype ANXA11-expressing iPSC-derived neurons for evidence of TDP-43 related changes using immunocytochemistry (Fig. 4A). Expression of P93S ANXA11 resulted in substantial loss of nuclear TDP-43 staining in iPSC-derived neurons (*mean* = 0.185 vs 0.386, *P* < 0.0001; Fig. 4B). Through direct interactions with intronic regions of pre-mRNAs, TDP-43 functions as a splicing repressor, and functional loss of nuclear TDP-43 causes abnormal splicing products termed cryptic exons.^31^ Two of the most-characterized cryptic-exons related to TDP-43 loss of function include those found in STMN2 and UNC13A mRNA transcripts (Fig. 4C–D; Supplementary Fig. 2A-B).^32–35^ We developed a new hybridization chain reaction fluorescence in situ hybridization (HCR FISH) method to identify and quantify cryptic exons in STMN2 and UNC13A in fixed iPSC-derived neurons. Using our HCR FISH assay, we quantified cryptic exon abundance in iPSC-derived neurons expressing wildtype or P93S ANXA11. Expression of P93S ANXA11 increased STMN2 cryptic exon expression per cell by 3.5-fold (*mean* = 14.65 vs 52.55, *P* < 0.0001; Fig. 4E–F) and the ratio of cryptic UNC13A transcripts to native per cell by 37% (*mean* = 0.1554 vs 0.2131, *P* = 0.04; Supplementary Fig. 2C–D). These results indicate that expression of P93S ANXA11 in human neurons results in histological and functional loss of nuclear TDP-43, consistent with other previously described ANXA11 mutations associated with amyotrophic lateral sclerosis/frontotemporal dementia.

### The P93S variant alters the transcriptome and proteome of neurons and microglia

**Figure 5:**
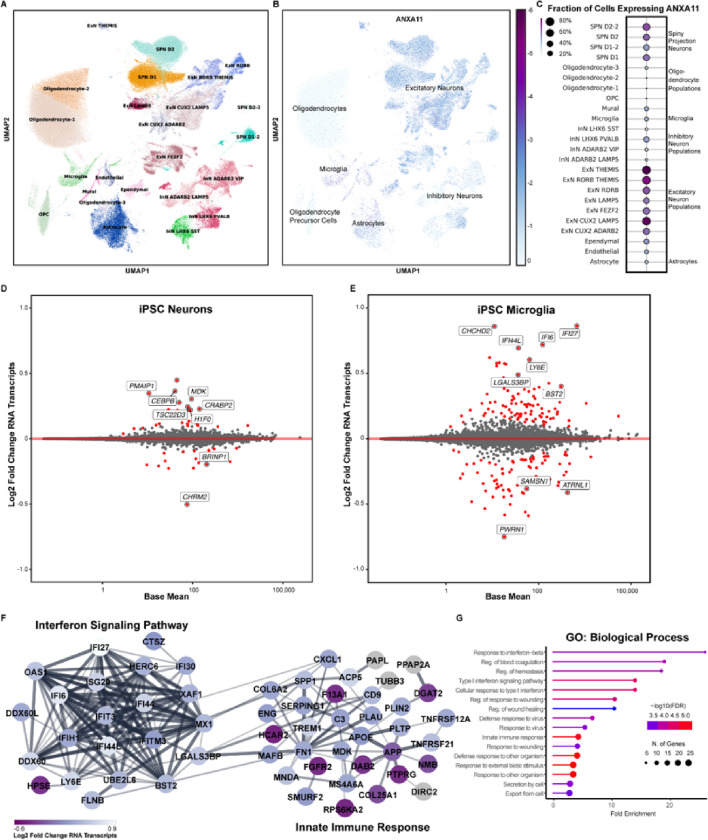
Transcriptomic signature of P93S. **(A)-(C) Expression map of ANXA11 in human brain cells. (A)** Single nuclei sequencing data from control brains demonstrating the two cell types that highly express ANXA11, microglia and neurons. Unsupervised clustering of 34 cell types in the human brain. UMAP projections illustrating different cell types identified is shown on the left **(A)** and ANXA11 expression levels shown on the right **(B).** Scale represents log_2_ normalized average gene expression levels. Dot plot illustrating the mean normalized expression of ANXA11 by cell type **(C)**. Scale represents percentage of total cell population expressing ANXA11, with color representing mean normalized expression per cell type. (**D) and (E) Differential gene expression between wildtype and mutant ANXA11. (D)** and **(E)** Bland-Altman mean difference plots where each dot indicates a gene for which there are counted reads from scRNAseq in iPSC-derived neurons **(D)** and iPSC-derived microglia **(E).** The x-axis is average normalized counts and the y-axis is log_2_ fold change. Neuronal differential expression yields few significant genes related to transcriptional regulation and endocytic vesicles **(D)** while many more differentially expressed in microglia related to transcriptional regulation and endocytic vesicles **(E). (F) and (G) Differential microglial gene expression interferon signaling pathway. (F)** STRING diagram of differentially expressed microglial genes in the interferon signaling pathway and innate immune response. Colors indicate p_adj_. **(G)** Gene ontology terms for biological processes of microglial gene expression hits indicating interferon response and innate immunity. Dot size indicates number of genes in each GO grouping and color indicates −log_10_(FDR).

Though ANXA11 biology has been most carefully studied in neurons, its role in other cell types in the CNS has not been systematically evaluated. Mining an existing single nucleus RNA sequencing dataset of post-mortem human entorhinal cortex, middle temporal gyrus, putamen, and subventricular zone from cases with no known neurological disease, we found that neurons—in particular, excitatory neurons—and microglia were among the highest ANXA11-expressing cells in the CNS (Fig. 5A–C).^36^

Next, we profiled transcriptomic and proteomic responses to ANXA11 P93S expression in iPSC-derived neurons and microglia. We performed transcriptional analysis using single cell RNA sequencing. There were no significant pathways identified in neuronal transcriptomic results. However, individual review of the differentially expressed genes in the P93S sample primarily related to calcium signaling (e.g. *PDE4D, RCN1*, and *FSTL5*) cell adhesion and endocytic vesicles (e.g. *TENM2, CDH13, STDN2* and *CHRM2*) and transcriptional regulation (e.g. *SLC3A2, PCBD1, INSM1, H1F0, EIF1, CRABP2, CEBPB* and *TSC22D3*) (Fig. 5D). Interestingly, microglia exhibited a substantially greater differential gene expression response to ANXA11 P93S expression. GO term analysis revealed alterations in the interferon signaling pathway and innate immune response (Fig. 5E–5G).

**Figure 6:**
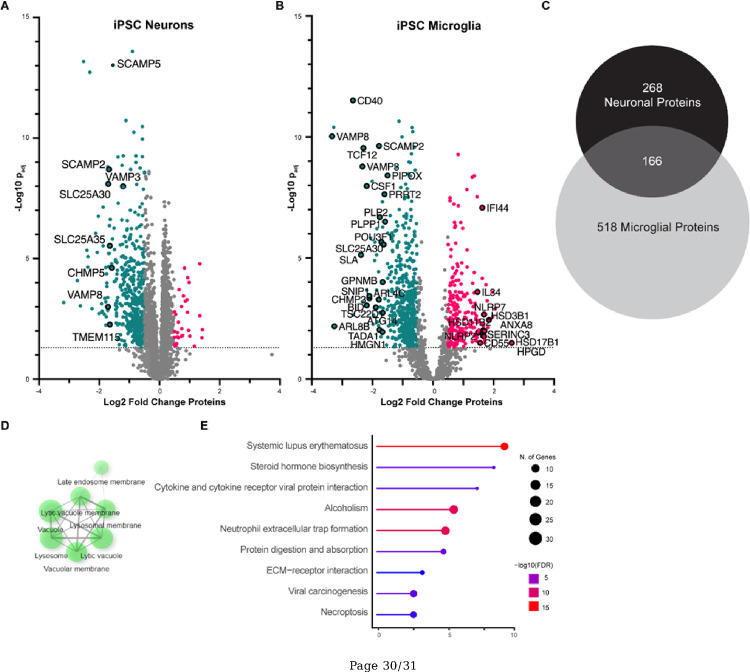
Proteomic signature of P93S. **(A)** Volcano plot of proteomic changes in neurons identifying significant changes in proteins involved in vesicle transport and SNARES, corroborating functional assays indicating that P93S disrupts proper functioning of ANXA11. **(B)** Volcano plot of proteomic changes of mutant compared to wildtype microglia implicating inflammatory pathways, as well as transcriptional regulation and vesicular transport. Lipid metabolism protein perturbations seen are indicative of microglial dysfunction. Red dots indicate upregulated proteins (FC>2) and blue dots indicate downregulated proteins (FC<−2). Dotted line demarcates a −log_10_ of p_adj_ value of 1.3. **(C)-(E) Unique microglia proteomic signature. (C)** Venn diagram of significant differentially expressed proteins in neuron (n = 434) compared to microglia (n = 684). Significant is defined as padj>0.05. **(D)** Non-overlapping significant microglial proteins cluster in the lysosomal cellular component gene ontology. **(E)** Representation of KEGG terms of non-overlapping significant microglial proteins. Dot size indicates number of genes in each GO grouping and color indicates −log10(FDR).

We further characterized how ANXA11 P93S expression altered the proteome of neurons and microglia using shotgun proteomics. Expression of ANXA11 P93S did not result in any statistically significant increases in protein expression in neurons after correction for multiple comparison (Fig. 6A). However, down-regulated neuronal proteins included ribosomal proteins, those involved in vesicle transport, endosome cargo processing, and SNARE proteins (e.g. VAMP8, SCAMP2, SLC25A30 and 35, TMEM115 and CHMP5). These proteomic changes in neurons are consistent with functional assays indicating that the P93S mutation disrupts the tethering function of ANXA11.

Proteomic analysis of iPSC-derived microglia revealed substantial alterations in the setting of ANXA11 P93S expression (Fig. 6B). Down-regulated proteins broadly grouped into three categories: inflammatory pathways (e.g. PLPP1, CD40, SLA, BID, CSF1, GPNMB, PLP2 and PIPOX); transcriptional regulation (e.g. SNIP1, HMGN1, POU3F1, TADA1, TCF12, and TSC22D1); and lysosomal SNARE proteins involved in vesicular transport (e.g. VAMP3, VAMP8, PRRT2, SLC25A30, ATGI4, ARL4C, and ARL8B). Expression of P93S in microglia up-regulated proteins associated with the innate immune response including CD55, NLRP7, IFI44, and SERINC3. Additionally, up-regulation of proteins HSD3B1, HSD11B2, HSD17B1 and HPGD involved in lipid metabolism pathways including prostaglandins synthesis, glucocorticoids and steroids, suggest microglial dysregulation due to this mutation.^37^ These findings indicate potential dysregulation of immune pathways, consistent with measured abnormalities in the cellular and protein biomarkers of innate immunity in P93S patient biofluids. Expression of ANXA11 P93S caused substantially more changes in protein expression in microglia (684 proteins) than in neurons (434 proteins) (Fig. 6C). No significant pathways were identified in dysregulated proteins specific to neurons. Dysregulated proteins specific to microglia clustered in lysosome-related cellular component gene ontology terms (Fig. 6D) and several significant KEGG pathways were identified, including relating to systemic lupus erythematous—an autoimmune condition—and cytokine signaling (Fig. 6E). Taken together, these results reveal that both neurons and microglia may be involved in disease pathophysiology in patients with ANXA11 P93S variants, and that the transcriptional and proteomic responses to expression of this variant are more pronounced in microglia.

## Discussion

This study describes a novel clinical presentation associated with mutations in ANXA11, including characterization of a previously unreported variant of uncertain significance and establishes its pathobiology. ANXA11 has previously been associated with amyotrophic lateral sclerosis and frontotemporal dementia. More recently, reports expanded the presentation to include an inclusion-body myopathy-like multisystem proteinopathy as well as semantic variant primary progressive aphasia.^10,38,39^ We describe a series of cases presenting with corticobasal syndrome, further expanding the phenotypic spectrum of ANXA11 mutations. We use disease-relevant cellular models to confirm the pathogenicity of the P93S variant through the demonstration of both loss of proper ANXA11 function and pathologic TDP-43 changes as well as identifying cell-specific bioactivity of the variant through comparative omics.

We previously reported on the function of ANXA11 as a tether between RNA granules and lysosomes during axonal transport, which is disrupted by disease-causing mutations.^3^ Prolines are unique among amino acids for their cyclical side chain, conferring rigidity in intrinsically disordered domains and solubility to decrease the likelihood of aggregation.^40–42^ These properties may be altered with the otherwise relatively conservative amino acid change to a serine. Moreover, amino acid sequences within intrinsically disordered domains, while not necessarily conserved in invertebrates, are highly conserved among vertebrates (Fig. 2B–C).^43,44^ Thus, the loss of a proline residue within this region may have an outsized effect on protein structure and function that is not captured by typical *in silico* prediction models. Despite contradictory annotations using *in silico* modeling, the specialized function of prolines in low complexity domains as well as the conserved amino acid sequence in position 93 and rarity of this variant suggest that the P93S is a deleterious mutation, underscoring the limitations of existing tools to accurately classify VUS. Interrogating the known function of ANXA11 in iPSC-derived neurons, we show that this mutation results in disrupted tether function and involvement of TDP-43 pathways, supporting the pathogenicity of this VUS. Therefore, despite conflicting results from *in silico* predictions, we were able to confirm pathogenicity of this VUS using cellular models; we exhibit the utility of these models to inform the classification of variants, especially in disordered protein regions where current predictive models fail.

In neurons, ANXA11 is involved in axonal transport whereas its role in microglia is not well known. We use unbiased proteomics to detect differential effects of this ANXA11 variant in neurons and microglia. Our cellular models indicate that immune dysregulation is a major affected pathway and microglia may have an important role in driving pathology. These findings also provide an important link relating to observations in human disease. In our patient biofluids, we find expansion of CD4 + and CD8 + T-cell populations present in both central and peripheral compartments and elevated CSF IgG, indicating increased blood-brain barrier permeability and loss of immune system homeostasis. There is growing recognition of the role of inflammation and immune dysregulation in neurodegeneration, including the development of a pro-inflammatory milieu in the central nervous system.^45^ Genes implicated in both monogenic neurodegenerative disease as well as risk genes are related to microglia and immune pathways, such as *APOE, TREM2*, and *TBK1*, and many of which were also identified in our microglial transcriptomic and proteomic data. Like the observations seen in this family, clonal expansion of CD4 and CD8 positive T-cells have been described in amyotrophic lateral sclerosis type four and frontotemporal dementia patients, further supporting a link between immune dysregulation and disease.^46–49^ Previously published ANXA11 cases report white matter changes and we similarly find a unique imaging pattern in this cohort consisting of central predominant atrophy with T2 FLAIR white matter hyperintensities.^10^ Potential explanations for this include primary oligodendroglial dysfunction, primary axonal injury or microglial-based inflammation. However, expression profiling indicates that ANXA11 is not highly expressed in oligodendroglia, but is found in microglia. Moreover, white matter has been shown to be particularly vulnerable to inflammation and microglia are the major immune effector cell within the central nervous system.^50^ Thus, with the association of ANXA11 with sarcoid—a systemic autoimmune condition, our multiomic findings suggest that microglial ANXA11 contributes to disease and immune dysregulation, reflecting the importance of disease-relevant cellular models.

Low complexity domain sequences are often only conserved among mammals and specific residues have outsized importance such that even relatively conservative changes can disrupt normal function.^41,43,44,51^ In fact, several established disease-causing mutations in ANXA11 and other amyotrophic lateral sclerosis/frontotemporal dementia genes illustrate the limits of *in silico* modeling for mutations within low complexity domains (Fig. 2D). Accurate variant annotation is critical for its value in understanding biology as well as the diagnostics and therapeutic implications for patients. The discordant findings from current prediction algorithms highlight the shortcomings of this approach and emphasize the need for improved methodology to determine variant pathogenicity. We demonstrate the feasibility and power of cell-relevant iPSC-derived modeling systems to assess the bioactivity of a VUS, search for elements of human disease, and provide evidence of pathogenicity. The identification of a VUS in the context novel disease phenotypes, such as the one described here, in neurodegenerative disorders with high phenotypic variability pose a particularly vexing challenge. This challenge will only continue to grow with expanded access to genetic testing and widening disease spectrums in neurodegeneration, highlighting the critical need for improved methods to classify VUS.

In conclusion, we describe a family with a novel clinical presentation and a novel VUS in the amyotrophic lateral sclerosis/frontotemporal dementia associated gene, ANXA11. Through querying neurodegenerative disorders clinics, we establish corticobasal syndrome as part of the ANXA11 phenotypic spectrum. We also establish the pathogenicity of the P93S VUS by demonstrating decreased colocalization of mutant ANXA11 with lysosomes and a resultant decrease in neuritic RNA in iPCS derived neurons, indicating a loss of proper neuronal ANXA11 function. Pathogenicity of this variant is further supported by loss of nuclear TDP-43 and increased formation of cryptic exons in our cellular model. We establish the multiomic signature of the P93S variant, which supports the functional assay findings in neurons and reveals profound microglial changes related to immune pathways and interferon signaling, in addition to the known role in vesicular transport and transcriptional regulation. This sheds light on the potential role of ANXA11 in microglia and suggest that microglia play an important role in disease pathobiology, relating back to patient observations and human disease. Lastly, these findings show the power and promise of using generalizable techniques in cellular models to enhance variant annotation across genes, uncover new genotype-phenotype relationships, and provide insights into disease mechanism.

## Figures and Tables

**Table 1:** ANXA11 VUS cases and clinical presentations

Patient Number	Mutation	Age	First Symptom	Clinical Features	MRI Findings	CSF
1	P93S	60–77	Right leg spasticity	Bradykinesia, asymmetric spasticity, dystonia, apraxia, dysphagia, dysarthria	WMH Spinal WMH Central predominant atrophy Corpus callosum thinning	Elevated protein Elevated IgG and IgG index Negative Oligoclonal bands Elevated CD4 + CD8 + T-cells
2	P93S	50–79*	Right leg spasticity	Apraxia, dystonia, cortical sensory loss, alien limb	WMH Spinal WMH Central predominant atrophy Corpus callosum thinning	Elevated protein Elevated IgG Negative Oligoclonal bands Elevated CD4 + CD8 + T-cells
3	P93S**	Unknown-49	Lower extremity dysfunction	Tremor, dysarthria, dystonia	WMH	N/A
4	P93S**	Unknown-60	Lower extremity dysfunction	Spasticity, dysarthria, dysphagia, dystonia	N/A	N/A
5	G189E	51–66	Lower extremity spasticity	Dysarthria, dysphagia	WMH	Elevated IgG Matched serum and CSF oligoclonal bands
6	Y103H	59	Left arm apraxia	Corticobasal syndrome	Central predominant atrophy Corpus callosum thinning	N/A
7	R404W	N/A	N/A	Corticobasal syndrome	WMH Central predominant atrophy Corpus callosum thinning	N/A
8	S55L	N/A	Behavioral variant frontotemporal dementia	Parkinsonism	Central predominant atrophy	N/A
9	S55L**	N/A	Spasticity	Cortical sensory loss Dysphagia	N/A	N/A

WMH: T2 FLAIR white matter hyperintensities; N/A: not available

* Still alive

** Presumed mutation; no genetic testing available
